# Practical Notes and Formulæ

**Published:** 1881-12-20

**Authors:** 


### PRACTICAL NOTES AND FORMULÆ.

**Diarrhœal Disorders of Infancy.**—Dr. L. A. Davidson, of West Virginia, writes in the Medical and Surgical Reporter: I offer the following as a remedy in diarrhœal disorders of children, when the child has passed dentition, and diarrhœa has been protracted some time, through summer heat—

| | |
|---|---|
| Syrupi sacchari albi | ℥j, |
| Pulv. rhei | ʒj, |
| Sodii bicarb | ʒiss, |
| Pulv. columbæ | aa ʒij, |
| Pulv. pepsin, Americanæ | |
| Tinct. capsici | gtt. xxj |

One teaspoonful every other time the bowels move, in new, cold, alkaline milk (as can be had in rural districts.)—*Drug. Circular.*

**Aquarium Cement.**—The following is recommended by Dr. John Phin—

| | |
|---|---|
| Litharge | 3 parts, |
| White sand | 3 " |
| Plaster of Paris | 3 " |
| Resin | 1 part. |
| Boiled linseed oil, sufficient. | |

The solids are to be taken by measure in powder and mixed. As it sets rapidly the oil must not be added until it is wanted for use. It is better for being put into a mortar and pounded. It hardens in three days. It will hold glass firmly, and with it glass tanks may be made without frames, if the angles are well filled with cement. It is a kind of mastic, and could be used on brick.—*Drug. Circular.*

**English Hop Bitters.**—

| | |
|---|---|
| Orange peel | 2 ounces, |
| Calamus root | 1 ounce, |
| Burnet saxifrage root | 1 " |
| Hops | ½ " |
| Alcohol | 16 fl. ounces, |
| Water | 24 fl. " |
| Sugar | 4 " |

—*Druggists' Circular.*

**Spasmodic Dysmenorrhœa.**—

| | |
|---|---|
| ℞ Powdered valerian | ʒiii, |
| Laudanum | gtt. x, |
| Aquæ ferv | ℥viii. |

M. Sig. As a rectal enema in conjunction with baths and antispasmodics.—Prof. F. B. Fonsgreves, of Paris, in Medical Gazette.

**For Worms.**—

℞ Castor oil ............................ }

Spts. turpentine.......................... } aa 1 oz.

Tinct. myrrh............................ }

Worm seed oil.............................. 2 dr.

M. Sig. Give half a teaspoonful, with as much molasses, according to the age of the child, on an empty stomach, two or three times, half an hour apart.—*Clinical News.*

**Inflamed Hemorrhoids.**—

℞ Glycerine,.............................. 3 parts,
Gelatine.................................. 1 part,
Ex. bellad. vel opii........................ ½ grain.

The gelatine is melted in the glycerine, and suppositories are obtained of consistence for introduction into the anus. It should be introduced as deeply as possible.—[Med. and Surg. Rep.

**Styptic Colloid.**—The following instantly coagulates blood, forming a consistent clot, under which wounds will readily heal—

Collodion.................................. 100 parts.
Carbolic acid.............................. 10 "
Tannic acid............................... 5 "
Benzoic acid.............................. 5 "

Mix the ingredients in the above order.

**Repression of Beat Production.**—Quinine is used for this purpose. It should be administered in large doses. Three or four doses are sufficient. Digitalis produces a contraction of the arterioles—

℞ Quiniæ sulphatis......................... grs. x,

Tincturæ digitalis......................... mj or iss,

Acidi hydrochlorici dil.................... m v.

M. Sig. One dose; to be taken in one glassful of water.—*Ex.*

**Ointment for Scabies.**—Dr. H. G. McAllister, of McAllisterville, Pennsylvania, physician in charge of the Soldiers' Orphan School at that place, sends us the following formula, which he has found efficacious in curing scabies, with which the children of the school had been afflicted for a long time—

℞ Hydrarg bichloridi.......................... ʒij,

Pulv. capsici................................ ʒj,

Pulv. sulphur................................ ʒiv,

Adipis...................................... ℔iv.

M. Sig. Mix by gentle heat and keep stirring it constantly while cooling.—[Med. and Surg. Rep.

**Ointment.**—It is said that almost a perfect basis for ointments is made by mixing cold cream, made from oil of almonds, white wax and spermaceti, with cosmoline, in suitable proportions.

**For Habitual Constipation.**—

℞ Fluid ext. cascara sagrada .................... 1 oz.

Glycerine ............................... 1 oz.

Fluid ext. nux vomica ........................ 1 dr.

M. Sig. Teaspoonful night and morning.—[Peoria Medical Monthly.

**Headache.**—We select the following prescriptions for headache from a number collected from various sources by the Hospital Gazette.—[Med. News.

URÆMIC HEADACHE WITH DEFICIENT RENAL ACTION.

℞ Potass citrat ............................ ℈ j

Spts. juniperi ............................ ʒ j

Spts. æther. nitros ........................ ♏ xx

Decoc. scoparii ........................... ℥ j

M. Sig. This amount three times a day.

HEADACHE ASSOCIATED WITH GOUTY DIATHESIS.

℞ Liq. potass. ars ..............................

Liq. potassæ ........................... aa ʒ j

Tinct. colchici ........................... ʒ ij

Tinct. lavandulæ. co ....................... ℥ iij

Aquæ pura ............................... ℥ vj

M. Sig. A tablespoonful in a wineglassful of water twice a day after food.

DYSPEPTIC HEADACHE WITH FLATULENCE, ACIDITY AND PYROSIS.

℞ Sodæ bicarb ..............................
Bismuth subcarb ............................
Pulv. acaciæ ........................... aa ʒ j
Spt. amm. aromat .......................... ℥ ij
Syr. zingib .............................. ℥ iij
Aquæ puræ ............................ ad ℥ viij

M. Sig. Two tablespoonfuls three times a day, half an hour before food.

CONGESTIVE HEADACHE.

℞ Ammon. bromid ........................... ʒ j

Spt. amm. aromat .......................... ʒ ss

Aquæ puræ ............................... ℥ jss

M. Sig. To be taken on rising in the morning.

BILIOUS HEADACHE WITH FLATULENCE.

℞ Magnes. sulphat .......................... ʒ vj

Magnes. carbonat .......................... ʒ j

Tinct. lavand, co .......................... ℥ iij

Aqæ menth. pip ......................... ad ℥ viij

M. Sig. A sixth part to be taken early in the morning and repeated as may be necessary.

NEURALGIC HEADACHE WITH CONSTIPATION.

| | | |
|---|---|---|
| ℞ | Quiniæ disulph........................... | gr. xij |
| | Acid. sulph. dil........................... | ʒ ss |
| | Tint. ferri perchlor..................... | ʒ ij |
| | Spt. chloroformi......................... | ʒ ij |
| | Magnes. sulph............................ | ℥ jss |
| | Syr. zingiberis........................... | ℥ ij |
| | Aquæ.......................................ad | ℥ xij |

M. Sig. Two teaspoonfuls three times a day.

NEURALGIC AND NERVOUS HEADACHE MARKED BY GENERAL DEBILITY AND DEFECTIVE NUTRITION.

| | | |
|---|---|---|
| ℞ | Clacis hypophos.......................... | gr. 80 |
| | Tinct. ferri perchlor...................... | ʒ iij |
| | Quiniæ disulphat ......................... | gr. xvj |
| | Strychniæ.................................. | gr ss |
| | Spt. chloroform........................... | ʒ ij |
| | Syrupi...................................... | ℥ jss |
| | Aquæ puræ ad............................ | ℥ viij |

M. Sig. A tablespoonful three times a day in a wineglassful of water.

**Case of Gonorrhœa.**—Male, age 23, suffering from gonorrhœa, applied to me Gctober 12th for relief. He said the discharge appeared four days after exposure, and was very slight for the first three days. He had tried a remedy recommended by a friend, and at the end of seven days found himself very much worse, the discharge being quite profuse, thick and creamy. I gave him the following:

| | | |
|---|---|---|
| ℞ | Aqua camphora.......................... | |
| | Aqua dist............................aa | 3 ounces |
| | Acid boracic.............................. | 2 drachms |

M. Inject four times daily.

At the same time giving specific directions concerning his diet, habits of living, etc. October 17th, patient returned, the discharge had ceased, but he complained of strange itching sensation in the urethra." Gave him same injection, except adding 15 minims of carbolic acid to the six ounce mixture, also an alkaline diuretic. October 25th, returned again saying he was cured.—[Dr. MURDOCK, in Peoria Med. Monthly.

**Treatment of Hydrocele.**—Dr. T. L. Ogier, of Charleston, S. C., in American Bi-Monthly, gives his experience in the cure of hydrocele, which is prompt and painless. His method is to inject with a hypodermic syringe, one drachm of tincture of iodine into the sac, once in three days until the fluid is absorbed. The desired result is usually obtained in about ten or twelve days. The doctor's theory is that the injected iodine makes the fluid in the sac only sufficiently stimulating to cause the absorbents to absorb the fluid. —*Northwestern Lancet.*

